# Medical Experts

**Published:** 1870-07

**Authors:** 


***Medical Experts.***

A city paper, in view of this topic advances a few sensible ideas, which we hope will awaken interest in this most important matter, among the people. Any physician who has ever been on the witness stand as an expert must realize their essential truth. In the "hypothetical case" put to the witness, the lawyer, of course, always "supposes" the facts proved as he wishes them to be proved. Meanwhile the expert may positively know that the testimony in no degree warrants any such hypothesis.

We fervently trust that this is the true explanation of certain testimony (otherwise atrocious and disgraceful to the profession to the last degree) in the McFarland case in New York.

Our secular contemporary says:

"Several recent trials have exhibited the folly of the present system of examining experts in court, and especially physicians, in regard to the sanity of the prisoner, witness, testator, or other interested party. Under the present practice, a doctor is called by the prosecution and another by the defense, or, more frequently, several of these learned gentlemen by each party to the proceeding. The witness is examined, not by the court or the jury, but by the attorneys, and not on the evidence already adduced, but on the fruitful suppositions furnished by the lawyers. The physician who testifies in the case may not have heard a particle of the evidence before the court, but, if a prosecution witness, is asked: "If such and such be the case, is not this man insane!" Of course, he must answer in the affirmative. If he is called by the defense, on the other hand the form of the question is: "If so and so be true, can this man possibly be insane?" Of course, he replies in the negative.

The result of this mode of procedure may be, and indeed usually is, to array a formidable host of learned men, who go to work apparently to contradict each other in the most scandalously absurd manner. The poor juror, with the best intentions in the world, but with a not particularly acute penetration, and befogged more than usual by the shrewd manœuvering of the lawyers, says, with the rest of mankind, "When doctors disagree, who shall decide?" and then gives his verdict according to his latest impressions or to his private estimate of the relative reliability or honesty of the medical witnesses. The doctors, too, whose intentions may be quite as good, go out of court at least in a very ridiculous position, and often under the aspersion of perjury, or dishonesty, or incapacity.

"The practice of the courts should be changed in this matter. If the question be purely professional, and the result turn entirely upon the confirmed sanity or insanity of an individual, as is often the case, then the trial should be before a jury of experts, when consultation would not be simply a trial of obstinacy, but a comparison of scientific research that would be likely to lead to truth. If other interests enter into the trial, and it is not considered desirable to have a jury composed of one class of men, with similar habits and thoughts, then the testimony of experts should be taken after all the other evidence is in, and under the charge of the court. Under this practice, there would be less of the apparent confliction of testimony, confusion of juries, injustice in verdicts, and scandal among the doctors."

